# Advancements in metastatic spinal cord compression treatment: a narrative review of innovations, challenges, and future directions

**DOI:** 10.1097/MS9.0000000000004382

**Published:** 2025-11-25

**Authors:** Tirath Patel, Fathimathul Henna, Iman Sharif, Anaya Noor, Hafiza Tooba Siddiqui, Zarhaish Barkat-Ullah, Mahnoor Ishaque, Najia Ali Khan, Christopher Hanani, Abhishek Goyal, Nikhilesh Anand

**Affiliations:** a Department of Neurosurgery, Trinity Medical Sciences University School of Medicine, Kingstown, Saint Vincent and the Grenadines; b Department of Surgery, Dubai Medical College for Girls, Dubai, UAE; c Department of Surgery, Akhtar Saeed Medical and Dental College, Lahore, Pakistan; d Department of Surgery, Dow Medical College, Karachi, Pakistan; e Department of Surgery, Jinnah Sindh Medical University, Karachi, Pakistan; f Department of Surgery, Fatima Memorial Hospital College of Medicine and Dentistry, Lahore, Pakistan; g Department of Surgery, Khyber Medical College, Peshawar, Pakistan; h Department of Neurology, Henry Ford Health, Warren, Michigan, USA; i Department of Neurology, JFK University Medical Center, Edison, New Jersey, USA; j Department of Medical Education, University of Texas Rio Grande Valley, Edinburg, TX, USA

**Keywords:** artificial intelligence (AI), metastatic spinal cord compression (MSCC), minimally invasive surgery, personalized medicine, proton therapy

## Abstract

**Objective:**

To synthesize recent advancements in metastatic spinal cord compression (MSCC) treatment, evaluating their efficacy in improving neurological function, pain relief, survival, and quality of life.

**Background:**

MSCC is an oncologic emergency caused by the progression of cancer to the spine and is prevalent in approximately 3–5% of all cancer patients, with a relatively higher incidence of breast, prostate, and lung cancers. MSCC can lead to irreversible paralysis due to loss of vital sensory and motor functions, thus requiring an immediate diagnosis and treatment.

**Methods:**

A systematic PubMed search identified studies published over the past 5 years focusing on advancements in MSCC treatment. Applying the PICO criteria, 132 articles were retrieved, of which 38 met the inclusion criteria after rigorous screening.

**Result:**

Effective care of MSCC includes high-dose corticosteroids, surgery, and radiation therapy. Advancements such as robot-assisted and minimally invasive surgeries enhance surgical outcomes. Emerging technologies, including deep learning models for early detection and automated MRI analysis, improve diagnostic accuracy. Personalized medicine techniques, such as next-generation sequencing, enable the development of customized therapies tailored to individual tumor characteristics. Innovations such as bioengineered implants provide better imaging compatibility and reduce radiation scattering. Proton therapy delivers targeted radiation with minimal harm to adjacent tissues.

**Conclusion:**

Prompt, multimodal treatment is necessary, including corticosteroids, surgery, and radiation therapy to reduce symptoms and enhance patient outcomes. Despite advancements, challenges such as delayed diagnosis and limited access to specialized treatments persist. Precision medicine, artificial intelligence–based diagnostics, and easier access to clinical trials should be the main areas of future development. Preventing irreparable neurological impairments and improving patient quality of life requires a focus on palliative care and early intervention. Integrating innovative therapies with comprehensive supportive care in a patient-centered study is essential for enhancing neurological function, pain control, and overall quality of life.

## Introduction

**Figure 1.**
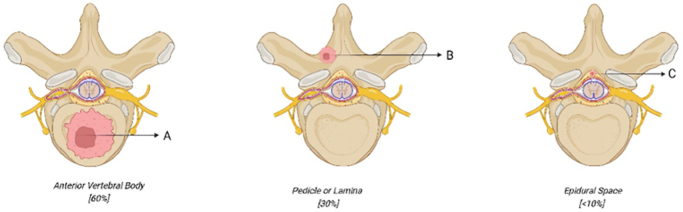
Metastatic involvement of spinal structures most commonly affects the pedicles and lamina (~60%), followed by the anterior vertebral body (~30%), while epidural space involvement is relatively rare (<10%).

Metastatic spinal cord compression (MSCC) is a recognized complication of cancer that typically occurs as an oncologic crisis. Spinal metastasis is prevalent in about 3–5% of all cancer patients, with a relatively higher incidence of 19% in breast, prostate, and lung cancers^[1]^. MSCC is an aggressive progression of cancer to the spine, marked by severe pain, neurological impairment, and a lower survival expectancy. Specifically, the thoracic spinal vertebrae are the first to be affected by MSCC, followed by the lumbar, sacral, and cervical spine^[2]^. The incidence of spinal metastases was slightly higher in men than in women, with a peak age range of 40–65 years. Epidural space involvement in spinal metastases occurs in less than 10% of all cases. Sixty percent of metastatic bone lesions are found in the anterior part of the vertebral body. In 30% of cases, cancer spreads to the pedicle or lamina^[3]^. The patterns of metastatic spread in the spine are depicted in Figure 1.

Persistent pain in the spinal cord and continuous discomfort that worsens with daily activities are typical signs of MSCC. Weakness and numbness in the lower extremities, as well as sensory deficits, are also crucial for identifying MSCC^[4]^. Every year, approximately 20 000 new patients with MSCC are diagnosed in the United States^[5]^. MSCC can lead to irreversible paralysis due to the loss of vital sensory and motor functions^[4]^. Diagnosis of MSCC is extremely important for prompt treatment and recovery. MRI is the first-line choice for diagnosis, offering a high sensitivity of approximately 93% and a specificity of 98% for metastatic tumors^[6]^. CT remains valuable, with 66% sensitivity and 99% specificity for diagnosis^[4]^.

There are multiple treatment options for MSCC, with the primary goals of reducing pain and maintaining spinal integrity. Treatment options include corticosteroids, surgical interventions, and radiation therapies^[4]^. Corticosteroids provide pain relief in MSCC and alleviate pressure on the spinal cord^[7]^. This study evaluates advancements in MSCC management, emphasizing their impact on clinical outcomes and the role of artificial intelligence (AI), personalized medicine, and novel therapies. Separation surgery is a new method that involves the surgical decompression of the spinal cord followed by stabilization. This approach allows for more comprehensive tumor removal while also providing a safe target for future radiation therapy. This results in the safe delivery of radiation doses to the targeted tumor site^[8]^. Patients with spinal instability are referred for surgical consultation followed by radiotherapy (RT) to improve treatment outcomes^[4]^. This study therefore evaluates recent advancements in MSCC management, focusing on their impact on clinical outcomes and emerging trends in AI, personalized medicine, and novel therapies.

This manuscript is made in compliant with the TITAN checklist to ensure transparency in the reporting of AI^[9]^.

## Methodology

### Literature search strategy

A comprehensive literature search was conducted to identify studies reporting advances in the treatment of MSCC. Four electronic databases: PubMed, Embase, Scopus, and the Cochrane Library were systematically searched for publications from 1 January 2018 to 28 February 2025.

The search strategy combined Medical Subject Headings (MeSH) with free-text keywords. The Boolean string applied was: (“Spinal Cord Compression”) AND (“Metastasis”) AND (“Treatment”). Search syntax was adapted to meet the indexing requirements of each database. In addition, the reference lists of included studies were manually screened to identify further eligible publications.

**HIGHLIGHTS**

- The most effective approach for metastatic spinal cord compression (MSCC) is a prompt, multidisciplinary strategy combining corticosteroids, surgery, and radiation therapy to prevent irreversible neurological damage.
- Innovations such as robot-assisted surgery, stereotactic body radiotherapy, and proton therapy are enhancing treatment precision and reducing harm to healthy tissue.
- Deep learning models are improving the accuracy of early diagnosis, while next-generation sequencing allows for customized, tumor-specific therapies.
- A strong focus on palliative care, including effective pain management and rehabilitation, is crucial for improving patients’ overall quality of life.
- Despite major advancements, significant challenges remain, including diagnostic delays, limited access to advanced therapies, and a scarcity of high-level evidence from large-scale randomized trials.
- The future of MSCC treatment lies in integrating precision oncology, artificial intelligence–powered diagnostics, and enhanced supportive care to improve patient outcomes.

### Eligibility criteria

The PICO framework guided the selection of studies. The population of interest included patients with MSCC. Interventions encompassed surgical treatment, RT, immunotherapy, and bone-targeting therapies, either individually or in combination. Comparators included no treatment, standard care, or alternative interventions. The primary outcomes of interest were neurological recovery, pain relief, overall survival, and quality of life.

Studies were included if they were peer-reviewed systematic reviews, meta-analyses, or narrative reviews, published in English between 2018 and 2025, and available in full text. Articles were excluded if they did not meet the PICO framework, addressed non-metastatic spinal cord conditions, or represented primary research designs [e.g., randomized controlled trials (RCTs), cohort studies, case-control studies, or case reports]. Further exclusions included conference abstracts, proceedings, editorials, commentaries, and letters, as well as non-English publications, duplicate records, and studies lacking sufficient relevance or outcome data.

### Study selection process

The initial search identified 7968 records. After duplicate removal using Zotero reference manager, titles and abstracts were screened independently by two reviewers. Any disagreements were resolved through discussion, and a third reviewer was consulted in cases of persistent disagreement.

Following title and abstract screening, studies that did not meet the eligibility criteria were excluded. Full-text review was then conducted to assess relevance and methodological quality. Ultimately, 38 studies were deemed eligible and included in the final synthesis.

**Figure 2.**
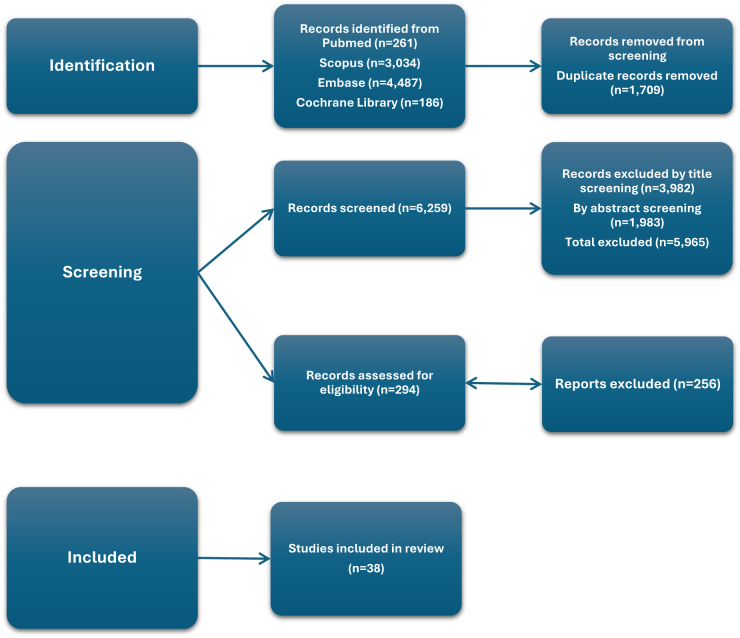
PRISMA-style flow diagram illustrating the article selection process, detailing the number of records identified, screened, excluded, and included, resulting in 38 articles included in the review.

The study selection process is illustrated in the PRISMA flow diagram (Fig. 2).

### Data extraction and synthesis

Data from the included studies were extracted into a structured proforma. Extracted variables included study design, intervention type, outcomes assessed, and key findings. Given the heterogeneity of study methodologies and outcome measures, a narrative synthesis was undertaken rather than a quantitative meta-analysis.

The strength and quality of the evidence were appraised according to the Oxford Centre for Evidence-Based Medicine (OCEBM) 2011 Levels of Evidence and cross-referenced with the GRADE framework. Table 1 further summarizes the evidence obtained from various study types included in the manuscript according to the Oxford Centre for Evidence-Based Medicine (OCEBM) or GRADE guidelines.

**Table 1:** Summary of the level of evidence for all included studies according to the Oxford Centre for Evidence-Based Medicine (OCEBM) or GRADE guidelines

S.No.	Study ID	Design	Sample size	Evidence
1.	Robson *et al* (2014)	Short review	NA	V
2.	Lawton *et al* (2019)	Review	NA	V
3.	Victor Tse *et al* (2024)	Education article	NA	V
4.	Boussios *et al* (2018)	Review	NA	V
5.	Kwok *et al* (2005)	Editorial	NA	V
6.	Perrin *et al* (2004)	Review	NA	V
7.	NICE (2023)	Clinical reference	NA	V
8.	Di Perna *et al* (2020)	Qualitative review	NA	V
9.	Loblaw *et al* (2003)	Retrospective cohort Study	121 435	III
10.	Esperança-Martins *et al* (2023)	Review	NA	V
11.	NICE (n.d.)	Clinical reference	NA	V
12.	Rades *et al* (2022)	Retrospective cohort Study	545	III
13.	Rades *et al* (2013)	Retrospective cohort Study	2029	III
14.	Gripp *et al* (2010)	Prospective cohort Study	216	II
15.	Patel *et al* (2013)	Cross-sectional Study	852	III
16.	Halperin *et al* (1985)	Review	NA	V
17.	Alshareef *et al* (2021)	Systematic review	5726	I
18.	Khan *et al* (2015)	Systematic review	NA	I
19.	Husain *et al* (2013)	Review	NA	V
20.	Fomchenko *et al* (2022)	Review	NA	V
21.	Kalfas *et al* (2001)	Review	NA	V
22.	Menta *et al* (2024)	Case series	7	IV
23.	Viswanathan *et al* (2012)	Retrospective report	95	IV
24.	Anselmetti *et al* (2010)	Review	NA	V
25.	Jensen *et al* (2002)	Review	NA	V
26.	Cotten *et al* (1996)	Retrospective observational study	37	III
27.	Calmels *et al* (2007)	Prospective observational study	52	II
28.	Barragán-Campos *et al* (2006)	Retrospective observational study	117	III
29.	Bhatt *et al* (2013)	Narrative review	NA	V
30.	Tseng *et al* (2008)	Retrospective study	57	III
31.	Chen *et al* (2009)	Retrospective Study	31	III
32.	Ofluoglu *et al* (2009)	Narrative review	NA	V
33.	Anselmetti *et al* (2009)	Prospective observational study	22	II
34.	Qian *et al* (2011)	Retrospective study	48	III
35.	Dudeney *et al* (2002)	Prospective Study	18	II
36.	Lieberman *et al* (2001)	Prospective case series	30	II
37.	Bouza *et al* (2006)	Systematic review	NA	I
38.	Goldvaser *et al* (2019)	Review	NA	V
39.	NICE guideline (2023)	Clinical reference	NA	V
40.	Rispoli *et al* (2022)	Retrospective study	257	III
41.	Xu *et al* (2021)	Retrospective observational study	39	III
42.	Fan *et al* (2021)	Retrospective Cohort Study	47	III
43.	Ong *et al* (2022)	Systematic review	NA	I
44.	Asano *et al* (2022)	Retrospective Observational Study	29	III
45.	Barzilai *et al* (2022)	Retrospective observational study	84	III
46.	Kiselev *et al* (2024)	Narrative review	NA	V
47.	Kendal *et al* (2025)	Systematic review and meta-analysis	NA	I
48.	Swanick *et al* (2023)	Retrospective study	10	III

### Ethical considerations

This review is based exclusively on previously published literature and did not involve direct patient participation or primary data collection. Therefore, ethical approval was not required for this review.

## Discussion

Effective management of MSCC as a serious complication of advanced cancer relies on a multidisciplinary, patient-centered approach that brings together the expertise of oncologists, radiotherapists, neurosurgeons, orthopedic surgeons, hematologists, and neuroradiologists. This collaborative effort has been shown to improve clinical outcomes^[10,11]^. This review explores the current treatment strategies, recent advancements, their impact on patient care, and future directions in MSCC management.

### Standard management of MSCC

MSCC is a neurological emergency treated by combining high-dose corticosteroids, RT, surgical intervention, and comprehensive rehabilitation, all of which should be initiated within 24 hours of diagnosis to prevent further neurological deterioration^[4,12]^. Corticosteroids, particularly dexamethasone, are routinely administered to reduce tumor bulk and spinal cord swelling, alleviate pressure, and enhance treatment outcomes^[7]^. Recent practice guidelines stress urgent intervention: immediate administration of high-dose corticosteroids (usually dexamethasone 16 mg/day) to decrease epidural tumor swelling^[13]^. MRI should be performed within 24 hours of clinical suspicion, and final treatment (surgery and/or RT) should ideally be initiated within that time frame^[14]^. In clinical practice, this translates into immediate neurosurgical consultation and radiation oncology consultation. If there are motor deficits, premature decompression (surgery or RT) is anticipated while steroids are continued. Rehabilitation and pain management are incorporated from the beginning^[14]^. By reducing tumor mass and swelling of the cord, dexamethasone may enhance neurological function, but is regarded as bridging therapy until local treatment is administered^[13]^. In general, the management is a team effort (neurosurgeon, oncologist, radiotherapist, and rehabilitation specialists) with steroids, spinal stabilization, and radiation on a priority basis^[13,14]^.

### Tailoring treatment: surgical and nonsurgical considerations

While the standard management of MSCC relies on a rapid, multidisciplinary response, overall management must align with patient prognosis and functional capacity, prioritizing individualized treatment strategies. Upfront decompressive surgery combined with RT provides significant benefits, particularly for patients expected to survive at least three months. However, many patients have a limited prognosis of less than three months, making them unsuitable for surgical intervention^[15]^. In such cases, best supportive care alone has been considered a reasonable option in several studies^[16–18]^. Fractionated external beam photon RT remains a cornerstone of MSCC management and plays a vital role in controlling tumor progression and preserving neurological function^[19]^. For patients with a predicted survival of ≥3 months, intensive local therapy is advocated. In reality, this will usually consist of decompressive surgery with spinal stabilization and then RT^[20,21]^. This hybrid technique (surgery + RT) is supported by prospective evidence demonstrating superior ambulation and pain outcomes compared with RT alone. Conversely, when life expectancy is very poor (<3 months) or performance status is poor, the priority turns to palliation: RT alone (typically single-fraction, e.g., 8 Gy ×1 for immediate pain relief) and care without operation is employed^[20]^. For the radiosensitive tumors and healthier patients, fractionated external-beam RT (e.g., 30 Gy in 10–12 fractions) is still the norm for lasting tumor control^[21]^. Stereotactic radiosurgery has the option of being utilized in individual cases (below). Consensus suggests that, when surgery is warranted, decompression should precede RT to alleviate pressure before local tumor control^[21]^. So an algorithm has arisen: patients with good performance and ≥3 months survival undergo surgery + RT, whereas others are treated with RT alone or supportive care^[20,21]^.

### Surgical interventions and minimally invasive techniques

Separation surgery is a novel approach that decompresses and stabilizes the spine, particularly in patients with significant spinal instability or neurological compromise. Advances in minimally invasive spine surgery (MISS) and posterior decompressive laminectomy with stabilization have improved outcomes by reducing morbidity and accelerating recovery. Minimally invasive surgery (MIS) has demonstrated fewer complications, reduced blood loss, and shorter operative time than open surgery (OS), making it the preferred choice for eligible patients^[22]^. Surgical intervention is typically reserved for patients with good functional status and a favorable prognosis, emphasizing the importance of individualized treatment. Consequently, minimally invasive procedures are increasingly adopted in eligible cases.

New surgical technologies have broadened the treatment options for MSCC. MISS methods, including percutaneous pedicle screws using muscle-splitting techniques, enable decompression and stabilization with significantly reduced blood loss and shorter length of stay than open surgery^[23]^. For instance, Fan *et al* demonstrated that freehand percutaneous screw fixation augmented by minimal decompression yields neurologic outcomes equivalent to open surgery but with quicker recovery^[23]^. Posterior decompression (laminectomy/hemilaminectomy) is still prevalent, but it is now practically always performed with instrumentation – isolated laminectomy without fusion is usually omitted because of instability^[20]^. Intraoperative navigation and robotics are increasingly used: image-guided systems and robot-assisted planning enable highly accurate screw placement and tumor resection, reducing complications^[24]^. For anterior column support, expandable titanium cages and advanced biomaterials (e.g., carbon-fiber or PEEK) allow customized fit; 3D-printed vertebral implants have shown dramatic improvements in fit and durability^[24]^. In fact, a recent meta-analysis found that patient-specific 3D-printed vertebral bodies have a 12-fold lower subsidence rate than conventional mesh cages^[24]^. Finally, image-guided vertebral augmentation is used for focal stabilization: percutaneous cement augmentation (vertebroplasty or balloon kyphoplasty) of metastatic vertebrae can rapidly reduce pain and strengthen bone. In one series of 39 patients with blastic spine mets, percutaneous vertebroplasty decreased pain scores (mean VAS from 4.3 to 2.4 at 3 months) and improved functional status^[25]^. MISS, navigation/robotics, expandable cages, and cement augmentation enhance MSCC reconstruction and pain relief.

### Advancements in radiotherapy

Recent advancements in MSCC management have emphasized the importance of precision and personalized treatment strategies. Stereotactic body RT (SBRT) has emerged as a significant innovation, offering highly focused, high-dose radiation in fewer sessions than conventional external beam RT (cEBRT)^[26]^. Spine SBRT specifically delivers high biologically effective doses to spinal metastases, aiming to optimize both tumor control and pain relief^[27]^. SBRT delivers high biologic doses to spinal tumors while minimizing exposure to the spinal cord and adjacent organs. Recent prospective data, including a 2024 phase II clinical trial of repeat SBRT for spinal metastases, showed 94% local control at 1 year, and an 83% overall pain response rate, and extremely low rates of myelopathy, supporting the role of SBRT even in the re-irradiation setting.^[28]^. SBRT is especially effective for oligometastatic disease or radioresistant histologies, as ablative doses can overcome tumor resistance. Furthermore, a meta-analysis published in 2024 comparing single-fraction SBRT versus multi-fraction SBRT regimens found similar local control, with multi-fraction regimens associated with lower rates of vertebral compression fractures, highlighting the importance of individualized fractionation strategies^[24]^. Evolving developments include adaptive RT (e.g., MR-guided online plan adaptation) that can cover for anatomical changes occurring during treatment. Proton therapy, currently under investigation for spinal metastases, utilizes the Bragg peak effect to minimize radiation exposure to adjacent organs. Planning research supports this benefit: for instance, passive-scattering proton plans for thoracic MSCC significantly reduced heart and esophagus doses compared to photon SBRT^[29]^. With an increase in proton centers, spine SBRT using protons could decrease toxicity, although clinical evidence is still emerging.

### Pharmacologic therapies

While local treatments like surgery and RT are crucial for direct tumor control and spinal stabilization, systemic therapies also play a vital role in the comprehensive management of MSCC by targeting the underlying disease and its effects on the bone.

Systemic agents for bone modification are central to MSCC prevention and palliation. Bisphosphonates (such as zoledronic acid) and the RANKL inhibitor denosumab both block bone resorption by osteoclasts. These medications inhibit osteolysis around metastases, reducing pathological fractures and other skeletal-related events (SREs). Clinically, denosumab is at least as efficacious as zoledronate: a recent meta-analysis demonstrated that denosumab significantly delays time to first SRE and reduces fracture risk more than zoledronate^[13]^. These drugs also mildly alleviate bone pain and can exhibit anti-tumor activity in bone. However, all antiresorptives carry risks, including hypocalcemia and jaw osteonecrosis, requiring careful monitoring. Anabolic bone treatments are a new frontier: for example, romosozumab (an anti-sclerostin antibody) highly favors new bone formation and is approved for severe osteoporosis. It is used experimentally for metastatic lesions, but the idea is intriguing – linking bone-building drugs with antiresorptives to enhance spinal stability. In conclusion, contemporary pharmacotherapy for MSCC focuses on osteoclast inhibition (bisphosphonates, denosumab) to prevent fractures and alleviate pain^[13]^, with anabolic strategies under exploration.

### Artificial intelligence and personalized medicine

AI and precision oncology are transforming MSCC management, enhancing early detection and treatment personalization. Machine learning algorithms have been constructed to aid MRI analysis of spinal metastases, such as deep-learning models, which can automatically segment and detect spinal lesions, and radiomics, which can identify which lesions will progress. In the preliminary stages, research shows “good performance” of AI models in classifying spinal tumors and distinguishing tumor progression from benign changes^[20]^. These instruments will soon be used to predict prognosis and plan treatment (e.g., to pick out high-risk patients or delineate SBRT targets). From the systemic therapy perspective, next-generation sequencing of spine biopsies is now commonly utilized. Notably, tumor sequencing of spine metastases shows very high concordance of critical mutations with the original tumor and with other metastases^[28]^. Hence, genomic analysis of spinal tumors can uncover targetable mutations (EGFR, BRAF, etc.) that direct targeted therapy. Secondly, immune checkpoint inhibitors have revolutionized the field: medications such as pembrolizumab and nivolumab have produced responses in metastatic cancers with bone metastasis. In a study of advanced NSCLC, pembrolizumab (frequently administered with denosumab) achieved objective responses in bone mets and disease control in 72% of subjects^[21]^. Biomarkers like PD-L1 expression, MSI status, and tumor genomics are now used to personalize systemic treatment for MSCC patients. Briefly, the age of personalized medicine (integrating AI-powered imaging, genomic profiling, and immuno/targeted treatments) is emerging in MSCC management.

### Challenges in MSCC management

Metastatic involvement of the spine is a frequent complication of systemic cancer progression and often leads to significant morbidity. Surgery and external beam RT remain the cornerstones of palliative treatment, which aims to preserve neurological function, alleviate pain, and maintain functional status. Recent advancements in image-guided techniques and stereotactic radiation delivery have improved local tumor control. However, recurrent or radiation-resistant diseases continue to pose a major clinical challenge with limited therapeutic options^[30]^. Recurrent or radio-resistant disease is challenging: tumors such as renal cell carcinoma or sarcomas can recur despite RT, and re-treatment with RT or surgery is of limited benefit and increased risk. Access to care is also problematic – not every center has immediate access to MRI and spine specialists, so diagnosis and treatment are delayed in under-resourced environments. In addition, patient factors are highly variable: some have several comorbidities or a very limited lifespan, so aggressive intervention is not appropriate.

Prognostic accuracy remains limited, complicating treatment decisions and emphasizing the need for individualized assessment. These factors complicate decision-making, often requiring individualized assessment. For instance, NICE emphasizes urgent MRI and treatment for all suspected MSCC^[14]^, but pragmatically, some patients slip through the net. Overall, tumor and health systems’ heterogeneity, along with the conflict between aggressive treatment and palliation, complicate MSCC management^[14,20]^. These challenges require the development of more accurate and effective therapeutic techniques.

### New therapeutic modalities

New treatments are emerging. Bioengineered implants, such as 3D-printed porous titanium vertebral cages, are designed to match each patient’s anatomy. These individualized implants allow for osseointegration and possess much lower rates of collapse compared with mass-produced cages^[24]^. Other biomaterials (osteoconductive scaffolds, bioactive coatings) are intended to promote bone fusion. Stem cell therapies, including mesenchymal stem cells and osteoprogenitor cells, are being explored for bone regeneration in metastatic defects, though clinical validation remains limited. In cancer, new immunotherapies for solid tumors are being created that could potentially be used for MSCC in the future: CAR-T cell therapies target tumor antigens, while BiTEs enhance immune responses by facilitating direct interaction between immune cells and tumor cells. Preliminary trials in HER2+ and B7-H3+ cancers suggest immunotherapy may complement MSCC treatment, though these approaches remain experimental.

### Innovations in neurosurgical techniques

Advancements in neurosurgical techniques have significantly enhanced the precision and efficacy of MSCC treatments. Image-guided navigation, robot-assisted spine surgery, and expandable vertebral implants have refined surgical accuracy, reduced complications, and accelerated recovery. Additionally, percutaneous vertebroplasty and kyphoplasty are increasingly used to stabilize pathological fractures associated with MSCC.

Image-guided spinal navigation replaces conventional intraoperative imaging, enhancing surgical precision and spatial orientation. Its application in the management of spinal metastatic disease has proven particularly beneficial for navigating complex anatomy and minimizing the risk of iatrogenic injury^[31]^.

Robotic-assisted spine surgery has further advanced surgical accuracy, improved screw placement, and facilitates the precise navigation of spinal lesions compared to traditional techniques. This technology holds promise for reducing operative time and minimizing postoperative complications^[32]^.

Vertebral body resection for metastatic spinal tumors often necessitates robust reconstruction to maintain spinal stability. However, patients with cancer frequently face challenges related to compromised bone quality due to chemotherapy, RT, and malnutrition. Expandable titanium cages have emerged as a durable reconstruction option, providing structural integrity and improved functional outcomes. Long-term studies highlight the benefits of expandable titanium cages while addressing potential complications^[33]^.

Percutaneous vertebroplasty (PVP) is a minimally invasive procedure that has proven highly effective in alleviating spinal pain in patients with osteoporotic vertebral compression fractures and metastatic vertebral disease^[34–43]^. Similarly, percutaneous kyphoplasty (PKP) is used to manage painful spinal metastases^[44]^. PKP is a radiologically guided technique in which a balloon is inserted into the vertebral body to create a cavity before injecting bone cement. This approach allows for the controlled deposition of high-viscosity cement at relatively low pressures, thereby reducing the risk of cement leakage and associated complications^[45–47]^.

In addition to surgical and radiation procedures, pharmacological interventions are vital in the therapy of MSCC. Bone-targeting agents, including bisphosphonates such as zoledronic acid and RANK ligand inhibitors such as denosumab, play a crucial role in reducing SREs. These agents help prevent vertebral fractures, spinal cord compression, the need for radiation to the bone, and hypercalcemia of malignancy, ultimately slowing MSCC progression in patients with bone metastases^[26,48]^.

A detailed overview of various innovations in the treatment of MSCC, along with their efficacy and clinical outcomes, is shown in Table 2. These include improvements in neurological function, pain relief, overall survival, and quality of life, and offer insights into the effectiveness of emerging therapeutic approaches.

**Table 2:** Comparing different interventions and their efficacy

First author	Technique/intervention	Conclusion
Boussios *et al*^[4]^	Dexamethasone prevents neurological decline, stabilizes the spine, and relieves pain, given as a loading and maintenance dose (IV or oral). Patients with spinal instability are assessed for surgery, followed by radiotherapy if required. As an option for non-surgical candidates or those with poor prognosis, single-fraction radiotherapy may be suitable for limited life expectancy, emphasizing pain relief, complication prevention, and functional independence.	Managing spinal instability requires a multidisciplinary approach. Corticosteroids prevent neurological decline, surgery is considered for instability, and radiotherapy is an option for non-surgical candidates. Rehabilitation plays a key role in pain management, complication prevention, and improving functional independence.
Halperin *et al*^[19]^	The study highlights fractionated external beam photon radiotherapy as a key treatment for malignant CNS diseases. Using conventional external beam radiotherapy, it delivers radiation in multiple sessions to optimize tumor control while minimizing harm to healthy tissue.	Fractionated external beam photon radiotherapy remains a cornerstone of MSCC management, playing a vital role in controlling tumor progression and preserving neurological function.
Rades *et al* 2022^[15]^	The intervention in this study is palliative radiotherapy for metastatic spinal cord compression (MSCC). Different regimens, including 1 × 8 Gy, 5 × 4 Gy, and 5 × 5 Gy, were evaluated to determine their effectiveness in preserving or improving motor function in patients with a life expectancy of ≤2 months. The study aims to identify patients who may still benefit from radiotherapy despite their limited prognosis.	The study underscores the importance of a tailored approach to palliative radiotherapy for MSCC patients with a limited prognosis. By identifying key prognostic factors, the scoring system aids in selecting patients who are most likely to benefit, optimizing treatment decisions, and avoiding unnecessary interventions.
Rades *et al* 2013^[16]^	The study focuses on developing a scoring system to guide treatment decisions for patients with MSCC. The intervention involves assessing prognostic factors to determine whether patients are better suited for best supportive care or single-fraction radiotherapy, ensuring appropriate treatment based on survival probability.	The scoring system effectively identifies MSCC patients with poor prognosis, helping clinicians avoid overtreatment and focus on appropriate care.
Gripp *et al*^[17]^	The intervention in this study is palliative radiotherapy for end-stage cancer patients. The study evaluates the adequacy of treatment, focusing on radiation regimens primarily consisting of at least 30 Gy with fractions of 2–3 Gy. It also highlights the underuse of single-fraction radiotherapy despite the limited survival of patients.	Palliative radiotherapy in end-stage cancer patients was often prolonged, with many spending a significant portion of their remaining life in treatment without meaningful benefit. Unrealistic survival estimates led to extended regimens instead of shorter, more appropriate approaches, resulting in suboptimal patient care.
Patel *et al*^[18]^	This study examines the use of palliative radiotherapy in the final month of life, analyzing its frequency, timing, and potential impact on terminally ill patients. It evaluates whether the treatment provides meaningful palliation or survival benefits and considers the implications for optimizing end-of-life care.	Palliative radiotherapy near the end of life offers minimal benefit and should be carefully weighed against hospice care to avoid unnecessary treatment burdens.
Husain *et al*^[27]^	The study focuses on spine stereotactic body radiotherapy (SBRT) for spinal metastases, which delivers high biologically effective doses to improve tumor and pain control. It compares SBRT to conventional palliative radiotherapy, examines its integration with surgery, and highlights associated risks.	Spine stereotactic body radiotherapy offers a targeted approach with potential benefits but also carries risks not seen with conventional radiotherapy. Careful patient selection and risk assessment are essential to optimize outcomes.
Fomchenko *et al*^[30]^	The study explores palliative treatments for spinal metastases, including surgery and radiotherapy, alongside emerging targeted molecular therapies, chemotherapy, and immunotherapy. These newer treatments have shown improved responses in cancers like breast, lung, melanoma, renal cell, prostate, and thyroid, offering additional options for managing recurrent or radiation-refractory disease.	Integrating novel systemic therapies with existing treatment approaches can enhance disease management, aid surgical decision-making, and support a more personalized, multidisciplinary treatment strategy for patients with widespread metastatic spinal disease.
Kalfas *et al*^[31]^	Image-guided spinal navigation is a technique used to improve the accuracy and safety of spinal surgery, particularly in the management of spinal metastases. This technique uses a computer workstation and an infrared camera to track the movement of surgical instruments in relation to the patient’s spinal anatomy. The system allows for real-time navigation and provides the surgeon with a 3D view of the spinal anatomy, enabling more precise placement of screws, decompression, and tumor resection.	Image-guided spinal navigation is a valuable tool in the management of spinal metastases, offering improved accuracy, safety, and efficiency in spinal surgery. This technique has the potential to reduce operative time, morbidity rates, and costs while minimizing the need for conventional intraoperative imaging.
Menta *et al*^[32]^	Interventions used are Robotic-assisted surgery, utilized to improve accuracy and navigation in spinal metastases surgery, and Pedicle screw placement, which is assessed using the Gertzbein–Robbins classification system. Instrumentation, i.e., the average of four spinal levels instrumented per patient and Surgical fixation that is used to alleviate pain, preserve neurological function, and ensure mechanical stability.	Robot-assisted surgery is safe and effective at treating spinal metastases despite the high risk associated with it.
Viswanathan *et al*^[33]^	The techniques used in this study are vertebral body resection, reconstruction with expandable titanium cage, and spinal stabilization.	The use of an expandable titanium cage for spinal reconstruction in spinal metastases is safe and provides multiple benefits.
Anselmetti *et al* 2010^[34]^	The interventions in this study are vertebroplasty and kyphoplasty	Vertebroplasty is the preferred intervention in the majority of cases. However, kyphoplasty is recommended in certain conditions like large vertebral defects and spinal tumors.
	These techniques are used for vertebral Compression, fractures, and spinal tumors, according to this study.	
Jensen *et al*^[35]^	This study shows that percutaneous vertebroplasty is a minimally invasive procedure used for treating osteoporosis-related fractures and metastatic vertebral lesions.	Vertebroplasty shows promise for treating spinal diseases and tumors, particularly in the North American region.
Cotten *et al*^[36]^	The intervention in this study is percutaneous vertebroplasty.	Pain relief can occur even with incomplete lesion filling, and cement leaks into surrounding tissues rarely cause clinical problems.
	This study explores the efficacy of percutaneous vertebroplasty for spinal metastases and myelomas. A CT scan was performed 1–8 hours after methyl methacrylate injection to evaluate lesion filling and potential cement leakage into surrounding tissues, with results compared to clinical follow-up outcomes.	
Calmels *et al*^[37]^	This study evaluated the efficacy of percutaneous vertebroplasty in treating osteoblastic and mixed spinal metastases.	Vertebroplasty is effective in providing pain relief for osteoblastic and mixed spinal metastases, with satisfactory analgesic efficacy and acceptable clinical complication rates.
Barragan–Campos *et al*^[38]^	This study investigates the complications associated with percutaneous vertebroplasty using polymethylmethacrylate cement in patients with spinal metastases.	Despite some complications, PV is considered a safe approach for metastases to the spine. Systemic issues were linked to intravascular leakage, and local issues were thought to be tied to cement or needle-related irritation.
Bhatt *et al*^[39]^	Bhatt *et al* discussed recent and upcoming non-invasive and minimally invasive methods for the treatment of spine metastases, such as stereotactic body radiotherapy, vertebroplasty, kyphoplasty, radiofrequency ablation, and percutaneous fixation to relieve pain and stabilize the spine.	Minimally invasive and non-invasive techniques effectively manage pain, enhance spinal stability, and increase quality of life with less morbidity than open surgery. Progress in these methods continues to broaden treatment possibilities for metastatic spine disease.
Tseng *et al*^[40]^	Tseng *et al* studied minimally invasive vertebroplasty as a therapeutic intervention for pain due to spinal metastatic tumors. The technique is done by injecting bone cement into the fractured vertebrae to stabilize the fractures and alleviate pain with minimal surgical trauma.	Vertebroplasty is successful in relieving pain and stabilizing the spine in metastatic spine disease patients. It is a safe procedure with a minimally invasive nature and a lower complication rate, which makes it useful for palliative care.
Chen *et al*^[41]^	Chen *et al* investigated percutaneous transpedicular vertebroplasty with polymethyl methacrylate (PMMA) for the treatment of pathological spinal fractures. This minimally invasive technique stabilizes compromised vertebrae and relieves pain.	Vertebroplasty with PMMA is an effective treatment for pain relief and the restoration of spinal stability in patients with pathological fractures. It is a safe and effective alternative, especially for patients with metastatic spine disease or osteoporosis-related fractures.
Ofluoglu *et al*^[42]^	Ofluoglu examined minimally invasive techniques for treating spinal metastases, focusing on vertebroplasty, kyphoplasty, radiofrequency ablation, and percutaneous fixation. Vertebroplasty and kyphoplasty use bone cement to stabilize fractures and reduce pain, while RFA destroys tumor tissue through heat. Percutaneous fixation provides structural support with minimal soft tissue disruption, reducing the need for open surgery.	These minimally invasive approaches effectively relieve pain, restore spinal stability, and improve quality of life with lower surgical risks and faster recovery, making them ideal for palliative care in metastatic spine disease.
Anselmetti *et al* 2009^[43]^	Anselmetti *et al* examined temperature variations during bone cement hardening in percutaneous vertebroplasty. The research quantified *in vivo* temperatures to evaluate possible thermal injury to adjacent tissues during the hardening of the cement.	The study confirmed that while bone cement polymerization generates heat, the temperature remains within safe limits, minimizing the risk of thermal injury. These findings support vertebroplasty as a safe and effective procedure for stabilizing pathological spinal fractures.
Qian *et al*^[44]^	Qian *et al* evaluated kyphoplasty for treating malignant vertebral compression fractures caused by spinal metastases. The procedure involves inflating a balloon to restore vertebral height before injecting bone cement to stabilize the spine and relieve pain.	Kyphoplasty effectively reduces pain, restores vertebral height, and improves spinal stability in patients with metastatic spine disease. It is a safe and minimally invasive option with low complication rates and significant functional benefits.
Dudeney *et al*^[45]^	Dudeney *et al* studied kyphoplasty for treating osteolytic vertebral compression fractures in multiple myeloma patients. The procedure involves balloon inflation to restore vertebral height, followed by a bone cement injection for stabilization and pain relief.	Kyphoplasty is an effective and minimally invasive treatment for myeloma-related vertebral fractures, providing significant pain relief, improved spinal stability, and better quality of life with low complication rates.
Lieberman *et al*^[46]^	Lieberman *et al* evaluated kyphoplasty for treating painful osteoporotic vertebral compression fractures. The procedure restores vertebral height using balloon inflation before injecting bone cement to stabilize the fracture and reduce pain.	Kyphoplasty provides significant pain relief, improves spinal alignment, and enhances mobility in patients with osteoporotic fractures. It is a safe and effective minimally invasive treatment with low complication rates.
Bouza *et al*^[47]^	Bouza *et al* conducted a systematic review assessing the efficacy and safety of balloon kyphoplasty for treating vertebral compression fractures. The review analyzed clinical outcomes, pain relief, functional improvement, and complication rates.	Balloon kyphoplasty is an effective and safe procedure for vertebral compression fractures, providing significant pain relief, spinal height restoration, and functional recovery with a low risk of complications.
Goldvaser *et al*^[48]^	Goldvaser and Amir reviewed the role of bisphosphonates in breast cancer therapy, focusing on their use in preventing bone metastases, reducing skeletal-related events, and improving bone density in patients with breast cancer.	Bisphosphonates play a crucial role in breast cancer management by strengthening bone, reducing fracture risk, and potentially improving survival in certain patient groups. They are an essential component of treatment for bone metastases and osteoporosis in breast cancer patients.

### Current challenges and limitations in MSCC management

This narrative review has potential limitations, which should be acknowledged. The literature on MSCC is widely heterogeneous with differences in study designs, patient populations, cancer sub types, and outcome measures, which make direct comparisons challenging. In addition, RCTs are limited in this area, and most of the current evidence in the literature is derived from retrospective cohorts, case series, or single-arm trials, which present a higher risk of bias. Moreover, long-term data on functional recovery, quality of life, and longevity after various treatment modalities are scarce, limiting insight into the durability of therapeutic results. In addition, variances in reporting standards across studies, including inconsistent usage of valid scales for neurological or pain outcomes, limit the overall quality and generalizability of the findings. Lastly, publication bias cannot be entirely excluded, as negative or inconclusive studies are less likely to be published. These weaknesses underscore the critical need for large, prospective, multicenter randomized trials with standardized, comparable outcome reporting and longer follow-up to provide more substantial guidance for clinical practice.

### Strengths of existing evidence

The evidence supporting MSCC treatment is flawed, with some significant gaps. The majority of studies comprise small retrospective series or single-center experience with heterogeneous patient groups^[20]^. There is a sparse amount of randomized trials; aside from the standard decompression trial by Patchell *et al*, most evidence is derived from observational cohorts. There is variation in the outcome measures (motor function, pain, quality of life) and a short follow-up period. Thus, recommendations are presently based more on expert consensus than on high-level evidence. Meta-analyses of SBRT and surgery highlight this problem: they note high variability in dosing, fractionation, and inclusion criteria across studies. In short, there are no large, definitive RCTs to guide many MSCC decisions, and long-term efficacy/safety data are sparse^[20]^. This underlines the need to interpret existing studies cautiously.

### Future directions in MSCC treatment

MSCC treatment is evolving toward precision medicine, minimally invasive techniques, and enhanced supportive care. AI is expected to play a crucial role in early detection, prognosis prediction, and personalized treatment planning. Targeted therapies, including immune checkpoint inhibitors and molecular-targeted agents, offer promising potential to enhance tumor control while minimizing the need for aggressive interventions. Advances in radiation therapy, such as proton beam therapy and adaptive radiotherapy, aim to provide more precise tumor targeting with reduced damage to the surrounding tissues. Similarly, robot-assisted spine surgery and bioengineered implants are expected to improve surgical accuracy, reduce recovery time, and enhance spinal stability.

Beyond curative treatment, optimizing supportive care is essential. Early integration of palliative care, enhanced rehabilitation programs, and improved pain management strategies will significantly improve the quality of life of patients with MSCC.

Additionally, bone-targeting therapies, including anabolic agents and stem cell research, may strengthen vertebral integrity and facilitate spinal reconstruction. Expanding clinical trials and refining standardized guidelines will be critical in advancing MSCC management, ensuring broader access to innovative treatments, and improving patient outcomes globally.

**Figure 3.**
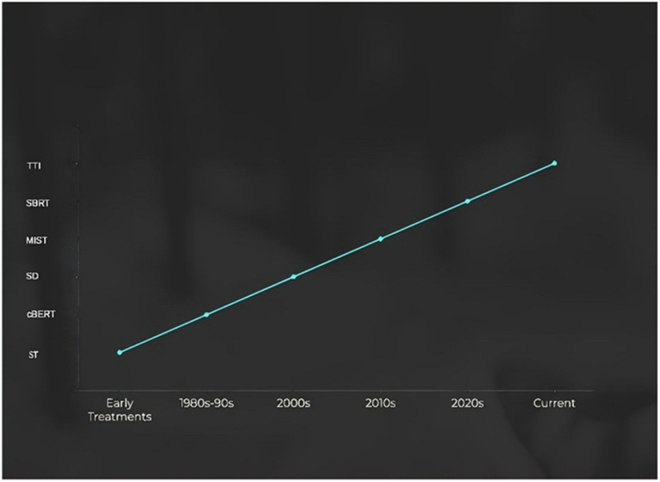
This illustration showcases the major advancements in therapeutic strategies, encompassing surgical techniques, various radiotherapy methods, and systemic treatments. It highlights the evolution of these approaches aimed at enhancing patient outcomes and overall quality of life.

MSCC treatment has evolved from steroid-based management in the 1980s–1990s to surgical decompression in the 2000s, minimally invasive techniques in the 2010s, and precision therapies like SBRT and proton therapy in the 2020s. The 2000s saw a rise in surgical decompression, while the 2010s introduced minimally invasive techniques, such as vertebroplasty. In the 2020s, SBRT and proton therapy improved precision. The current era focuses on targeted therapies and immunotherapies, which offer promising potential for tumor control. Figure 3 illustrates this evolving treatment trend.

Looking ahead, several trends will enhance MSCC care, including precision oncology, which tailors treatment based on tumor-specific genetic and molecular characteristics. Whole-genome analysis of spinal tumors (or liquid biopsies) will probably direct targeted and immune-based treatments. AI integration will streamline workflows by accelerating MRI analysis and optimizing radiation therapy planning. Adaptive RT (e.g., MRI-guided linacs) might make on-the-fly plan modifications feasible in the middle of treatment. Proton and heavy-ion therapy provide a chance to increase tumor dose with sparing of normal tissues. On the supportive care side, there is increasing recognition that early palliation and rehabilitation optimize outcomes. In cancer overall, research demonstrates that incorporating symptom management and rehab from diagnosis onward improves quality of life, an idea that needs to be translated into MSCC. Lastly, there is a need for coordinated research efforts, including prospective clinical trials, spine tumor registries, and international guidelines, to ensure uniformity of care. Specifically, a series of trials of new combinations (e.g., SBRT + immunotherapy) and technologies (adaptive RT, new implants) is urgently needed. In general, the management of MSCC in the future is multimodal, individualized treatment based on sound evidence and patient-specific care^[20]^. The future of MSCC management lies in personalized medicine, AI-driven treatment optimization, and novel therapeutic combinations. Continued research and broader access to advanced therapies are essential for enhancing MSCC survival and quality of life.

## Conclusion

MSSC occurs when cancer spreads to the spine, resulting in excruciating pain, neurological dysfunction, and reduced life expectancy. The management of MSCC includes a multidisciplinary approach combining high-dose corticosteroids, surgical procedures, RT, and systemic therapies to reduce pain, prevent additional neurological complications, and improve quality of life. SBRT allows for accurate tumor targeting, whereas minimally invasive surgery, robotic-aided surgery, and expandable vertebral implants improve surgical accuracy and recuperation. Targeted therapies, such as immune checkpoint inhibitors and bone-targeting therapies, improve disease management. Despite these advances, numerous issues persist, such as delayed diagnosis, research gaps, and limited access to advanced treatments. Future efforts should prioritize expanding access to innovative therapies, AI-driven treatment optimization, and clinical trials. Integrating innovative treatment with patient-centered approaches will help patients with MSSC achieve even better outcomes.

This review highlights key advancements in MSCC management while acknowledging existing challenges and areas for future research. However, it must also be acknowledged that there are some limitations in the current literature. Most of the studies included in this review were either retrospective in nature or small cohort studies, which limit the strength of their conclusions. The large amount of heterogeneity in study design, patient populations, and outcome measures presents a further challenge to conducting a formal comparison or even meta-analysis. Moreover, RCTs remain scarce, particularly for newer surgical techniques and AI-driven diagnostics. Longitudinal studies on post-treatment quality of life and functional independence remain scarce. This should remind us of the necessity for greater quality, standardization, and longitudinal data in this field of study.

## Data Availability

No new data were generated or analyzed during the study. Data sharing is not applicable to the article as it is a narrative review.

## Research registration unique identifying number (UIN)

Not required because it is a narrative review.
